# Adversarial vulnerability assessment of vision language models for healthcare

**DOI:** 10.1093/bib/bbaf631.004

**Published:** 2025-12-12

**Authors:** Rintaro Shiroshima, Kazuhiro Takemoto

**Affiliations:** Department of Bioscience and Bioinformatics, Kyushu Institute of Technology, Iizuka, Fukuoka, Japan; Department of Bioscience and Bioinformatics, Kyushu Institute of Technology, Iizuka, Fukuoka, Japan

## Abstract

**Background:**

Vision language models (VLMs) are increasingly integrated into medical workflows for diagnostic support and clinical decision-making. While recent studies have demonstrated susceptibility of proprietary VLMs to prompt injection attacks in medical contexts [1], the security landscape of domain-specific medical VLMs remains largely unexplored. This study comprehensively evaluates the vulnerability of multiple VLMs to both prompt injection and adversarial perturbation attacks [2], investigating white-box attacks on MedGemma and black-box transfer attacks across medical-domain and proprietary models.

**Methods:**

We conducted systematic vulnerability assessment using medical images with histologically confirmed malignant lesions spanning multiple modalities: CT, MRI, ultrasound, pathology, endoscopy, and dermatology (n = 18 cases, 3 per modality). For prompt injection, we embedded malicious instructions within text prompts and visual elements. For adversarial perturbations, we used Projected Gradient Descent and optimization-based methods. White-box attacks utilized full model access to MedGemma, while black-box attacks employed transfer-based methods using surrogate models (OpenCLIP, BiomedCLIP, BLIP).

**Results:**

MedGemma achieved the lowest prompt injection vulnerability (38% ASR), followed by Claude 4 Sonnet (48%), GPT-5 (57%), and Claude 4.1 Opus (69%), suggesting domain-specific medical training enhances resistance. For adversarial perturbations, white-box attacks on MedGemma exceeded 80% ASR. Black-box transfer attacks showed varying vulnerability: GPT-5 (44%), MedGemma (37%), Claude 4.1 Opus (17%), and Claude 4 Sonnet (6%). Vulnerability rankings differed notably between attack modalities.

**Conclusions:**

This study provides the first comparative security assessment across medical-domain and proprietary VLMs. Results reveal complex vulnerability patterns with no single model providing universal robustness across different attack vectors. These findings emphasize that robust medical AI security requires comprehensive, multi-layered defenses targeting both text-based and image-based attack vectors, with model-specific threat considerations for medical applications.

**References:**

1. Clusmann J. et al. ‘Prompt injection attacks on vision language models in oncology.’ Nature Communications 2025;16:1239.

2. Hirano H., Minagi A. and Takemoto K. ‘Universal adversarial attacks on deep neural networks for medical image classification.’ BMC Medical Imaging 2021; 21:9.

